# Expanding the framework of attention in posttraumatic stress disorder: Commentary on Clauss et al. (2025)

**DOI:** 10.1002/jts.70009

**Published:** 2025-07-24

**Authors:** Janne L. Punski‐Hoogervorst, Avi Avital

**Affiliations:** ^1^ Behavioral Neurobiology Lab, Department of Occupational Therapy University of Haifa Haifa Israel

## Abstract

This commentary expands on Clauss et al.’s (2025) meta‐analysis of attention control training (ACT) for posttraumatic stress disorder (PTSD) by situating ACT and related interventions within a broader framework of attentional functioning. Although ACT and attention bias modification (ABM) show promise in targeting specific attentional processes, both neglect key domains, such as divided attention and multisensory regulation, which are often impaired in PTSD. Drawing on neuropsychological and neuroimaging findings, we highlight the need for the application of a multidimensional model of attention that accounts for the complexity of trauma‐related attentional dysregulation. Future interventions should integrate a wider range of attentional components to improve clinical relevance and effectiveness.

The recent systematic review and meta‐analysis by Clauss et al. (2025) on attention control training (ACT) for posttraumatic stress disorder (PTSD) offers a valuable synthesis of emerging work in cognitive interventions targeting attentional dysregulation in adults with PTSD. As the field continues to search for scalable, neuroregulation‐focused treatments that complement or enhance traditional trauma‐focused therapies, ACT stands out as a theoretically grounded and low‐burden approach. Although Claus et al. (2025) make a compelling case for ACT's potential, their article invites discussion about what current attention‐based interventions may be missing.

When attention is conceptualized as a multidimensional system—consisting of the basic components of focused, sustained, and selective attention and the higher‐order components of alternating and divided attention (Petersen & Posner, 2012)—it becomes clear that both ACT and its sister intervention, attention bias modification (ABM), only target a subset of the broader attention function. ACT emphasizes top‐down regulation, primarily targeting sustained attention and aspects of executive control, such as the ability to alternate attention across stimuli in a goal‐directed manner. ABM, in contrast, seeks to recalibrate more automatic, bottom‐up attentional processes—especially orienting, focused, and selective attention—by training individuals to redirect their initial attentional response away from threat‐related stimuli.

However, when evaluated through a comprehensive attentional framework, both interventions reveal significant blind spots: Neither approach addresses divided attention, which is often impaired in PTSD and crucial for navigating complex, multitasking environments. Research has consistently shown that individuals with PTSD experience difficulties switching and distributing attention across concurrent tasks, as reexperiencing can involuntarily capture attention and interfere with the ability to divide attention across tasks. In our comprehensive review encompassing 49 neuropsychological studies on attention in PTSD, 61.2% of the studies showed significant correlations between PTSD symptoms and attention deficits, with 20.4% indicating that larger attention deficits predicted more severe PTSD symptoms (Punski‐Hoogervorst et al., 2023). Neuroimaging studies further support the presence of generalized attention deficits in PTSD: Altered activation has been observed in major attention‐related brain networks, including the dorsal and ventral attention networks and the salience network, even when participants engage in emotionally neutral tasks (Ely et al., 2023). These findings point to a neurobiological underpinning of attentional dysregulation in PTSD that extends beyond fear conditioning and threat vigilance.

Moreover, current ACT and ABM protocols are visually oriented and, therefore, neglect the multisensory attentional regulation deficits observed in individuals with PTSD. Notably, sensory gating (i.e., the neurological mechanism that filters out redundant or irrelevant stimuli) is often impaired in PTSD. This may be related to the previously noted reduced divided attention, which normally engages sensory gating mechanisms to maintain performance and prevent overload. For example, studies have demonstrated that individuals with PTSD exhibit reduced P50 sensory gating, indicating difficulties in suppressing repetitive stimuli (Javanbakht et al., 2011). This impairment may contribute to heightened sensitivity and hypervigilance to various sensory inputs, including auditory, visual, and tactile stimuli.

Although ABM focuses on reducing a consistent bias toward threat, other research suggests that variability in attention bias—rather than direction—may be a more meaningful marker of PTSD‐related attentional dysregulation. Iacoviello et al. (2014) found that attention‐bias variability (ABV) was more strongly associated with PTSD symptoms than static bias scores. ABV reflects instability in attentional control mechanisms, with attention fluctuating unpredictably toward and away from threat cues. This pattern mirrors the clinical presentation of PTSD, where individuals often oscillate between hypervigilance and avoidance. High ABV has been linked to impaired top‐down control and may serve as a more sensitive indicator of attentional dysfunction in PTSD than conventional attention‐bias metrics. These findings suggest that future attention‐based interventions might benefit from targeting executive attention stability rather than attempting to normalize directional bias alone.

These gaps raise important questions about the validity and generalizability of ACT and ABM in their current designs. Interventions that target narrow cognitive processes in decontextualized lab settings may fail to translate into functional improvements in the real world, where attentional demands are fluid, multimodal, and emotionally embedded. Future research would benefit from adopting a comprehensive model that integrates all attentional subtypes. In addition to advancing intervention design, it is equally critical to develop systematic assessment approaches that evaluate the full spectrum of attentional functioning in trauma‐exposed individuals. Identifying specific attentional deficits, whether in sustained, selective, divided, focused, or alternating attention, can inform individualized treatment planning and ensure that interventions are targeted to the most affected domains. Such a precision‐based approach may optimize outcomes, especially for individuals whose attentional difficulties extend beyond threat‐related attention bias. This expanded framework would better capture the complexity of trauma‐related attentional dysregulation and could inform the development of more effective and personalized intervention strategies.
